# Medical Society of the County of Albany

**Published:** 1872-11

**Authors:** F. C. Curtis

**Affiliations:** Secretary


ART. III.—*Medical Society of the County of Albany. Annual Meeting Nov. 12th,* 1872.

Reported by F. C. Curtis, M. D , Secretary.

The annual meeting of the Albany County Medical Society was held at the City Hall, in Albany, Dr. JOSEPH LEWI, President, in the chair. The Treasurer made his report, showing the Society to be in a flourishing financial condition. Dr. J. P. BOYD, of the Board of Censors, reported for membership the names of Drs. R. A. Starkweather, J. H. Lagrange, and G. L. Ullman, who were elected. Drs. Zeh, of Berne; and Gallup, of Knox; Fenelly, of W. Troy; and Felix Weidman, of Westerloo, were proposed for membership.

Dr. LEWI then made the annual address, as President, on the subject of Idiopathic Peritonitis. After alluding to traumatic and puerperal peritonitis, he continued: The above named kinds of peritonitis are especially favored by the different authors, with minute description of their nature, symptoms, and treatment, leaving for the *idiopathic,* or, as some call it, the rheumatic peritonitis, only a little space, under the pretense that this malady, contracted by a cold, is very rare, and hardly ever attacks persons in general good health.

Although the last mentioned variety of peritonitis is not as frequent as inflammation of other serous membranes, especially the pleura, I venture to maintain that peritonitis, especially in childhood, say from two to fifteen years of age, is of more frequent occurrence than pericarditis, and, from causes to be stated, more fatal than pleurisy. I will also state here that a twenty-six years' practice has taught me that this malady presents to a young practitioner more difficulties in forming a diagnosis, and adopting a mode of treatment, and more embarrassment in giving satisfaction as to prognosis, than any other inflammation.

Sometimes there are no premonitory symptoms, but in most cases a slight or violent chill will compel the otherwise well appearing individual to retire from his avocation. After six or twelve hours the inflammation will, in very distinct cases, manifest itself by an intense fever, frequent pulse, high temperature, and a more or less violent pain on one or the other side of the abdomen, which side or spot will be very sensitive to the touch, so much so that the pressure of the bed clothes will be intolerable to the patient, who lies still, immovable on his back, his lower extremities drawn up. He talks little, and in a low tone. After a shorter or longer time the abdomen becomes bloated and filled with gas, which crowds against the diaphragm to such an extent that the lungs are compressed and impeded in their action, respiration becoming difficult and frequent. Constipation and vomiting generally accompany these symptoms of acute peritonitis. In fatal cases all these symptoms increase in intensity and violence; the abdomen becomes hard and tympanitic; the lungs and heart become so compressed that the respiration is very laborious; the agony of the patient is terrible. At last he becomes apathetic and delirious, the pulse smaller and more frequent; the skin is covered with a cold sweat, and the patient dies. In a great many cases the disease does not reach such a deplorable height. The symptoms after having culminated, the intolerable pains and difficulty of breathing grow gradually milder and less, and the patient recovers after the lapse of a few days or weeks. And in some instances, even after the malady has assumed all the bad features described above, it makes a retrograde movement, the symptoms come to a stand still, exudation of the infiltrated blood takes place, an abscess forms, the contents of which either discharge through the walls of the abdomen by nature's efforts, or a surgical interference, or they penetrate into the adjoining rectum or bladder, whence they are expelled with the fæces or urine, and the patient may recover entirely, or may be troubled temporarily, or for the remainder of his life, with the results of this extraordinary termination of the disease.

So much for the regular process of cases of idiopathic peritonitis, as described by most authors, and known to all practitioners.

The management of such cases of peritonitis has been, as late as twenty-five years ago, strongly anti-phlogistic. Blood-letting by venesection and the application of leeches, mercurial preparations internally and externally, scanty nourishment and the mildest of drinks in the inflammatory stage; absorbents internally, and blisters externally, in the stage of exudation, has been the treatment of authors of former times. This treatment has been almost universally abandoned as irrational and pernicious in general; yet, although it has been demonstrated that by promoting the peristaltic motion of the bowels the symptoms of inflammation will be aggravated, and that loss of blood will add so much to the general debility resulting from the disease itself; and although we must admit that vast numbers of the human race have suffered an untimely death by this course, my long experience and close observation dictate to me to say here, that there are exceptional cases of peritonitis where the application of leeches, and even venesection, is not only permitted, but imperatively demanded to save the life in some cases, and to bring on a speedier cure in others. If a person extraordinary in development and plethoric in appearance is, after exposure, etc., seized with all the above described abdominal symptoms to such a degree as to bring, within twenty-four hours, his pulse to 120–130, the temperature to 103°, and his respiration to 40–50; if congestion of the brain, delirium and a cyanotic appearance of the face should accompany these symptoms, a practitioner is in my opinion not only justified, but morally obliged to resort to blood-letting. I have seen in a large number of cases the most beneficial results from it. The circulation and respiration improved so much that the inflammation took its natural course, and was in time conquered by less heroic medicines; and it is to be presumed in these cases, or a majority of them, the violent onset would have destroyed life before the disease had fairly developed, and before any other measure could have made any impression on the so disturbed system. But with the exception of blood-letting in such exceptional cases, and counter-irritants in the later stages of the disease, all the appliances belonging to the antiphlogistic and depleting mode of treatment ought to be relinquished for all time to come.

In a great many, and as my experience tells me, in a majority of cases of idiopathic peritonitis, the course of its development is irregular, and therefore not easily to be distinguished from other complaints of the abdomen; and if the practitioner does not take heed and is overhasty in forming a diagnosis, the patient's life may be endangered, or even destroyed, with the reputation of the physician. In such cases the patient becomes indisposed and morose, loses his appetite, and complains occasionally of a dull, annoying pain in the abdomen. Aversion to all kinds of solid food and an inclination to vomit satisfy him, or his neighbor, that his "stomach is out of order," and a good strong physic is taken. The remedy generally distresses the patient more or less; he tries, and after a while succeeds, in throwing up the same, as well as the contents of the stomach, with a good portion of bile. Being relieved of the distress, but exhausted by the exertion, he imagines that he feels better, but soon after complains of the pain all over the abdomen: at this stage only, even in better classes of society, the physician is sent for. The patient explains that he is not sick, only a little out of order for a few days back, that he was constipated, and took medicine for it, which was not strong enough, and that a strong laxative to remove the bile, will probably be the best for his case. The physician examines the case and finds a circumscribed space in the abdomen painful to the touch; that there is no expansion of the abdomen, no tympanitis, no fever; all the symptoms of the digestive organs correspond to the narrative of the patient, and he is very apt to act on the suggestion of the patient, and aggravate the case most decidedly. If the proper course is adopted, the patient will have the benefit of temporary relief under all circumstances, and if it be a mild case, he may recover within a few days. If it is a severe case the symptoms will develop themselves in the course of a day or two, so as not to leave any doubt in the mind of the physician as to the nature of the disease, and then it will be time to adopt the treatment of modern authors.

Peritonitis occurring in children under six years of age presents especial difficulties. Compared with other abdominal diseases at this age it is of very rare occurrence, and the parents having resorted to laxatives or very drastic vermifuges before the physician is called, the mischief is done to make the case a desperate one. The pain in the abdomen, before slight, has changed into a violent one. The physic did not evacuate the bowels, but caused them to swell up and be sensitive to the touch. Insatiable thirst, high fever and a tendency to vomit are the result of the harmless medicine given yesterday. If the physician is misled in this primitive stage, and induced to give purgatives, he will only find out his mistake when it is too late to correct it. To avoid the commission of such mistakes in similar cases, I would advise young practitioners to take the utmost pains in making the examination, to be reserved in expressing an opinion, and to adopt an expectant treatment. Warm applications in the form of wet flannel or poultices, small doses of opiates, mild drinks, a horizontal position and positive rest will in most of these cases accomplish a cure. In some cases it may assume a chronic course and require absorbents, counter-irritants and tonics; in others, the above semi-acute inflammation may after a few days develop into a violent acute inflammation, and require a more heroic treatment.

But under all circumstances, even if the diagnosis should turn out to be incorrect, and the patient should suffer from colic or worm fever, or the painful spot should develop into a muscular abscess, no harm will have been done, and no aggravation of the symptoms caused by the mode of treatment.

In all cases of suspected peritonitis, where the symptoms are not conclusive, the physician should call to mind all diseases that could be mistaken for it. Catamenial and uterine colic, hysterical pain in females, pains from indigestion, bilious colic, inflammation of the duodenum, stomach or liver, enteritis, retention of urine, worms and wind colic, present in some instances similar symptoms. The physician may examine whether the patient is at or near a menstrual period, or whether she is pregnant, or he may ascertain that these symptoms of peritonitis are due to the irregular functions of some afflicted digestive organ, and proceed energetically to relieve the patient at once. But if there remain a shadow of doubt in the mind of the physician about the existence of an inflammation in the peritoneum, he must content himself with the administration of such remedies as to relieve the patient of pain and distress, to give him necessary rest and comfort, and to keep his strength intact for the expected struggle.

Dr. LEWI closed his paper with a few words of congratulation to the society, and a brief allusion to the recent death of Dr. PETER VAN OLINDA.

On motion of Dr. QUACKENBUSH the thanks of the society were given to Dr. Lewi, and the paper referred to the State Medical Society for publication in their transactions.

Election of officers being next in order, Dr. SWINBURNE made remarks upon the lack of agreement in the society in the past, the overcoming this in a measure by the election of neutral officers during the past year, and the obligation the society was under to perpetuate this by moderation and concession on both sides for the present election. He offered a resolution that a nominating committee consisting of Drs. James McNaughton and J. P. Boyd, W. H. Bailey and J. V. P. Quackenbush be appointed, who should agree upon a ticket. After considerable discussion this was adopted by a *viva voce* vote of 31 to 29, and the committee retired for consultation.

Dr. DEVOL presented a ranular calculus of the size of a cherry stone, of light color and rough surface, which had been ejected from the sub-lingual gland of a patient. Dr. Sabin in the connection mentioned a case of his own, where he had removed a vesical calculus nearly as large as a peach stone from the urethra of a young woman. It had worked nearly down to the meatus, but was with difficulty removed.

The nominating committee reported through Dr. McNaughton that they were unable to agree upon a ticket. The election was then proceeded with and resulted as follows: President—A. VAN DERVEER. Vice President—A. W. SHILAND. Secretary—F. C. CURTIS. Treasurer—W. H. MURRAY. Delegates to State Medical Society—C. A. ROBERTSON, D. V. O'LEARY, A. FOWLER, H. MARCH.

Delegates to the American Medical Association and the board of Censors were continued the same as last year.

The society then adjourned.

